# Round Worms

**Published:** 1900-10-27

**Authors:** 


### ROUND WORMS.

Perhaps those of us who practice in England make a little too light of intestinal parasites. If, however, we turn to the experience of those who live in worm-infected countries we find that the presence of these parasites is looked upon as a very important matter, and is accepted as a probable cause of many symptoms and complications of disease which we should hardly think of referring to such an origin. Dr. Preston Maxwell,[1] of Changpoo in South China, says that, while in England the importance of infection of the intestinal tract with the round worm is hardly recognised owing to the comparative rarity of the disease, in the part of China where he is practising the case is very different, for he believes that, without any exaggeration, 99 per cent. of the inhabitants are infected with this parasite at one period or another of their lives. It is, he says, exceptional to find a child over the age of two who does not respond to a dose of santonin by the passage of one or more worms. On the other hand, tape worms and thread worms are very uncommon. By careful investigation he has made out that the infection arises from the eating of certain kinds of vegetables, particularly garlic and leeks, both of which are commonly manured with fæces, although usually only in the earlier stages of their growth. In some cases, however, it appeared probable that the origin of the mischief had been the eating of freshly gathered sugar-cane, which also is manured with fæces in the early stages. As to the number of round worms which may be passed at a single evacuation, the largest number he had himself counted was eighty-seven. "Eighty-five to ninety," he says, "is by no means a rare number; and one mother told me, in answer to my question 'How many?' that her son, a boy of eight, had had four large evacuations composed of worms." It would be very surprising if such an abnormal condition of the intestinal contents could continue long without producing symptoms, and, according to Dr. Maxwell, the symptoms produced are both definite and severe. First, in the case of the adult, he places a craving for food coming on about an hour after a good meal. This symptom is a reliable one, and the physician will find that about 99 per cent. of the patients in whom it is met with will pass worms under the influence of santonin. Another valuable sign is severe discomfort, of the nature apparently of colic, felt in the region of the stomach. Then there is a class of cases characterised by marked anæmia, cases in which the ordinary treatment for anæmia is utterly futile, but which, as soon as they have been throughly treated with santonin and have got rid of their unwholesome guests, improve with great rapidity. Turning to the case of children, one of the foremost symptoms is a large, flabby, protuberant abdomen, which in such a malarial district as that of South China, is apt to be mistaken by the layman for an enlarged spleen. Another much more common symptom is gastro-intestinal disturbance. Persistent stomach-ache, unconnected with the ingestion of unripe fruit, ought always to suggest to the practitioner in the East the probability of round-worm infection. This stomach-ache is often combined with alternate attacks of diarrhœa and constipation. Reflex symptoms are often present, such as convulsions, grinding of the teeth during sleep, nose-picking, preputial irritation, and night terrors. The latter often cease completely on the expulsion of the worms. Infected children also have a great tendency to sleep on their face, and they are often subject to great perversions of appetite. But as to appetite there is great variation, some being voracious and others having a positive disgust for food. As to diagnosis we have a sure and certain test, for the microscope enables us to say at once and without hesitation whether a patient has or has not the parasite, a small portion of the stool, or of the fluid evacuated under the influence of Epsom salts, being placed under the microscope and examined for the ova.

As to treatment the rule is plain: *give santonin.* But it must be properly administered, otherwise it may not only fail, but may produce most alarming symptoms of its own. Roughly speaking, all cases fall into two classes, the urgent and the not urgent.

In the former nothing answers better than a dose of an ounce of castor oil with four grains of santonin, to be taken at once, and to be followed after a week by one of the prescriptions now to be given for the cases which are not urgent.

In such chronic cases nothing answers so well as: santonin, gr. 1; hyd. c. cret., gr. 1; send six powders. One to be taken twice a day. This can be continued for several weeks if thought advisable.

Another prescription which has been found very useful is: santonin, gr. 1; hyd. c. cret., gr. 1; quininæ sulph., gr. 3. Send six powders, one to be taken twice a day.

It is often desirable to clear the intestine before giving the santonin, for which purpose calomel and jalap or colocynth and hyoscyamus may be used.

It will be often found in China that patients whose worms are being disturbed are affected with a sharp attack of fever.

[1] Journal of Tropical Medicine, October.

